# Review on Research Progress of Photoelectrochemical Biosensors

**DOI:** 10.3390/mi16111293

**Published:** 2025-11-19

**Authors:** Yu Zeng, Yuheng Wang, Yaqing Zhang

**Affiliations:** 1 Guangyang Bay Laboratory, Chongqing Institute for Brain and Intelligence, Chongqing 400064, China; 2 Department of Pediatric Orthopaedics, Xinhua Hospital, Shanghai Jiao Tong University School of Medicine, Shanghai 200092, China

**Keywords:** photoelectrochemical, biosensors, photoactive materials, signal amplification strategies

## Abstract

Photoelectrochemical (PEC) biosensors have emerged as a significant research focus in the fields of bioanalysis and medical diagnostics in recent years due to their high sensitivity, low background noise, and ease of miniaturization. This review summarizes the fundamental principles of PEC biosensors, recent advances in photoactive materials, signal amplification strategies, and typical applications. Photoactive materials serve as the source of the sensor signal and can achieve signal enhancement through strategies such as heterostructure construction, localized surface plasmon resonance (LSPR) effects, and defect engineering. PEC sensors have been widely applied in areas such as cancer liquid biopsy and pathogen detection; however, challenges remain, including material biocompatibility, anti-interference capability in complex samples, and lack of standardized platforms. Future development trends include the design of green and low-toxicity photosensitive materials, integration with microfluidic and wearable devices, and artificial intelligence-assisted signal analysis, which will promote the translation of PEC biosensors toward clinical applications and real-time detection.

## 1. Introduction

With the increasing global demand for early disease diagnosis, environmental monitoring, and food safety detection, developing detection technologies that are highly sensitive, low-cost, and easy to operate has become a research hotspot. Traditional detection methods, such as enzyme-linked immunosorbent assay [1], fluorescence analysis [2], and chemiluminescent enzyme immunoassay [3], although highly sensitive, generally suffer from complex operation, high cost, and reliance on specialized equipment. Therefore, there is an urgent need to develop novel detection platforms that meet the requirements for rapid, sensitive, and convenient analysis. To address the aforementioned issues, researchers have developed novel methods such as orthogonal surface-enhanced Raman scattering/field-effect transistor dual-mode detection [4] and electrochemical detection [5]. However, these approaches still face challenges, including complex signal coupling, high costs, and significant background signal interference. The photoelectrochemical (PEC) biosensor, as an emerging biosensing technology, holds promise as a new detection platform to meet the demands for rapid, sensitive, and simple analysis, due to its high selectivity, high sensitivity, low cost, ease of miniaturization, and simple operation [6,7].

Since Becquerel’s discovery of the photoelectric effect in 1839, photoelectrochemistry has gained widespread attention in various fields, including photocatalysis, photovoltaics, and PEC sensing devices [8]. In 1956, Clark invented the oxygen electrode, opening a new era in the field of biosensors. In 1962, Clark and others first proposed that biosensors are a new interdisciplinary technology, and subsequently, biosensors based on various techniques flourished, leading to the emergence of PEC biosensors [9]. Compared to traditional detection methods, PEC sensors generate signals through light excitation, enabling high-sensitivity detection with low background noise. This makes PEC sensors particularly prominent in complex samples, as they can reduce interference from non-target molecules or environmental factors. For example, a PEC biosensor based on a Z-type UiO-66/CdIn_2_S_4_ heterojunction and flower-like PtPdCu nanozyme demonstrated excellent specificity and stability in the detection of the HER-2 biomarker [10]. The principle is that photons are absorbed by a photoelectrode modified with photoactive materials, causing electrons to transition from the valence band (VB) to the conduction band (CB). These photogenerated carriers then undergo migration and recombination, producing an electrical signal. The analyte influences one or more of these processes by altering the properties of the photoactive material or the electrolyte environment, thereby triggering a PEC signal change for quantitative analysis [11,12].

However, despite significant advances in PEC biosensors, several challenges remain, particularly in the design of photoactive materials, improvement in carrier separation efficiency, and signal amplification strategies. In this regard, numerous review articles on PEC biosensors have been published. For instance, Zang et al. [13] reported the latest progress in PEC biosensing based on semiconductor nanomaterials. Devadoss et al. [8] provided new insights into signal amplification strategies for PEC biosensors. Bettazzi et al. [14] reviewed PEC gene sensors for detecting nucleic acid cancer biomarkers. More recently, Xiaoyun Xu et al. [15] focused on commonly used heterojunction types and defect engineering. Nevertheless, these reviews are either outdated or somewhat limited in scope.

Therefore, this review first introduces the working principles and advantages of PEC biosensors, and then systematically summarizes recent research progress, including advances in novel photoactive materials, signal amplification strategies, PEC sensor case studies, and integration with microfluidic technologies. Finally, we discuss potential future directions for PEC biosensor development.

## 2. Working Principle and Advantages of PEC Biosensors

**Figure 1:**
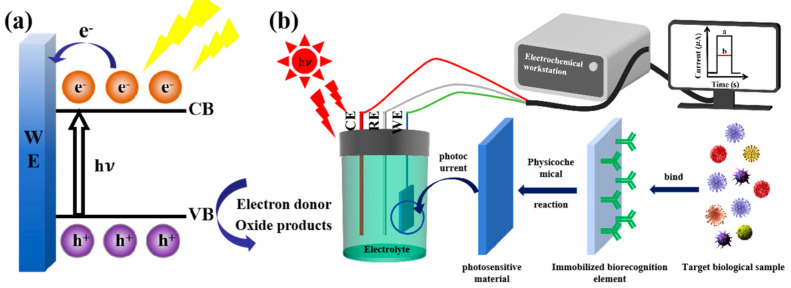
(**a**) Energy band diagram, (**b**) schematic illustration of the PEC biosensor.

**Figure 2:**
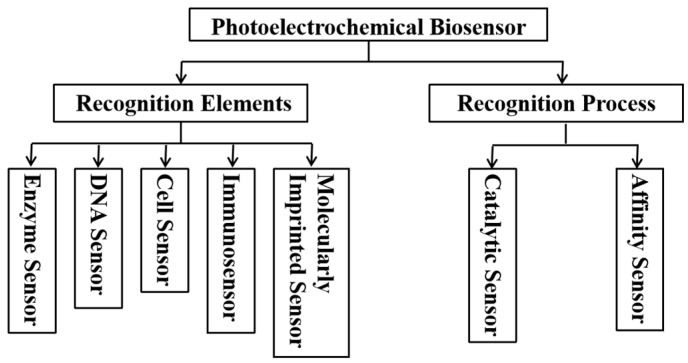
Diagram of the classification system of PEC biosensors.

The structure of the PEC biosensor, as shown in Figure 1, consists of the light source, electrode system, photoactive material, bio-recognition unit, electrolyte, and carrier transport. The sensors combine PEC analysis with specific biological recognition elements, such as antibodies, aptamers, or nucleic acid probes, to achieve efficient detection of target analytes. They typically employ a three-electrode system consisting of a working electrode, a reference electrode, and a counter electrode, where the working electrode serves as the site of interaction with the analyte. Photoelectric conversion involves complex dynamic processes, and the conversion efficiency directly determines the photocurrent signal of the sensor. According to the photoelectric effect based on band theory, the photoactive material is modified on the surface of the working electrode. When the photoactive material is exposed to light of a specific wavelength, electrons in the VB absorb photon energy and transition to the CB (as shown in Figure 1a), generating electron-hole pairs. These photogenerated carriers then migrate from the bulk to the surface, where they can only be effectively utilized when they reach the surface and enter the electrode substrate or electrolyte. Some carriers immediately recombine upon generation, while others recombine during their migration from the bulk to the surface. Some carriers recombine at the surface of the photoactive material due to the adsorption of electroactive species and the subsequent incomplete oxidation-reduction reactions, which consume the input energy [11]. The carriers that reach the surface of the photoactive material undergo oxidation-reduction reactions with electroactive species in the electrolyte, generating a photocurrent that can be detected by the external circuit [16]. Next, the biological recognition elements are immobilized onto the working electrode surface via physical adsorption, covalent bonding, or other methods [17,18]. When the target molecules in the sample bind to the recognition elements on the electrode, they induce physicochemical reactions that cause changes in the photocurrent [19,20]. This variation in the photocurrent is captured, amplified, and converted by specialized detection circuits or instruments, producing a measurable signal that reflects the concentration or presence of the target biomolecules in the sample (as shown in Figure 1b). By comparing the measured signal with a standard curve established using known concentrations of the target analyte, quantitative detection of the analyte can be achieved. The VB/CB energy levels of the photoactive material and the redox potential of electroactive species affect the migration and reaction rates of carriers. Based on this principle, improving the photon absorption and charge separation efficiency of the photoactive material, as well as accelerating electron transport as much as possible, are key points in enhancing sensor performance. The classification system of photoelectrochemical biosensors is shown in Figure 2. According to the classification based on recognition elements, the sensors can be divided into enzyme sensors, immunosensors, DNA sensors, cell sensors, and molecularly imprinted sensors. Based on the recognition process, they can be classified into catalytic sensors and affinity sensors.

PEC biosensors possess several notable advantages. Based on the PEC detection principle, they exhibit low background signals, and their performance can be further enhanced through strategies such as optimizing the illumination wavelength [21,22], improving the photoactive materials [6], and constructing heterojunctions. Moreover, their excellent compatibility allows them to handle a variety of sample types, including biological, environmental, and food samples. PEC biosensors can also be integrated with other techniques, such as microfluidics [23] and electrochemiluminescence [24], expanding their application range and functionality. As a result, they have attracted considerable scientific interest in fields such as bioanalysis, environmental monitoring, and food safety analysis [25].

## 3. Advances in Photoactive Materials

Photoactive materials play a crucial role in the construction of PEC immunosensors. The wide band gap of photoactive materials leads to low light absorption efficiency, while a narrow band gap may result in a higher recombination rate of photogenerated carriers, reducing the sensor’s sensitivity. Efficient carrier separation can reduce electron-hole recombination and enhance the photocurrent response. The chemical stability of photoactive materials directly determines the long-term performance of the sensor. Additionally, green materials have become a research focus due to their environmental friendliness, recyclability, and low toxicity. When selecting appropriate photoactive materials, it is important to consider not only traditional photoelectric properties but also their biocompatibility, environmental stability, and recyclability. For example, materials such as graphitic carbon nitride (g-C_3_N_4_), TiO_2_, and metal-carbon/nitride compounds (MXenes) are widely studied due to their non-toxicity, biocompatibility, and environmental friendliness. Therefore, when designing PEC immunosensors, it is essential to comprehensively evaluate the multiple properties of the photoactive materials. Currently, some representative photoactive materials, such as metal oxides, metal sulfides, graphitic carbon nitride, and quantum dots, have been widely applied in PEC immunosensors. Moreover, novel photoactive materials such as MXenes, metal–organic frameworks, and bismuth-based oxyhalides are gradually becoming research hotspots and demonstrate unique application value in PEC sensing technologies.

### 3.1. Metal Oxides

**Figure 3:**
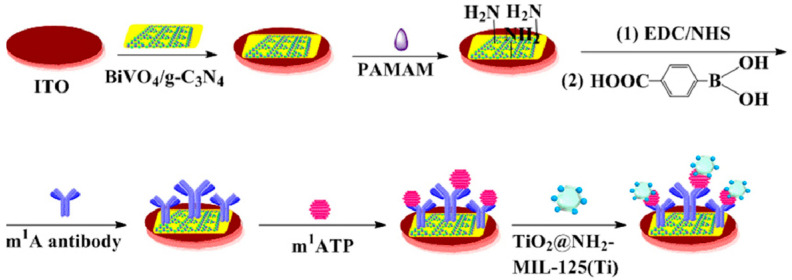
Schematic illustration of the PEC immunosensor structure [29].

Among commonly used semiconductor materials for photoelectrodes, metal oxides have attracted widespread attention due to their excellent chemical stability, suitable band positions, low cost, and tunable bandgap properties [26]. Among these materials, TiO_2_, as one of the most well-known photocatalysts, offers remarkable photocatalytic activity, low cost, non-toxicity, good thermal stability, and chemical stability [27,28]. Zhou et al. [29] designed a PEC immunosensor for the detection of N1-methyladenosine. As shown in Figure 3, the sensor employed a BiVO_4_/g-C_3_N_4_ heterojunction as the photoactive material, with polyamideamine and 4-carboxyphenylboronic acid serving as the antibody immobilization matrix for N1-methyladenosine, and a titanium-based metal–organic framework encapsulating TiO_2_@NH_2_-MIL-125(Ti) heterostructures for signal amplification. Due to the interaction between TiO_2_ and phosphate groups, TiO_2_@NH_2_-MIL-125(Ti) was captured after the immunoreaction between N1-methyladenosine and its antibody, and the enhanced PEC response further improved the detection sensitivity. In addition to TiO_2_, other metal oxides such as ZnO, WO_3_, CuO, PbO, Fe_2_O_3_, Co_3_O_4_, and BiVO_4_ also exhibit excellent biocompatibility and photocatalytic properties, making them highly promising for applications in PEC bioanalysis [8]. However, the wide bandgap of TiO_2_ results in low visible light absorption efficiency, thereby limiting its application in PEC [30].

### 3.2. Metal Sulfides

**Figure 4:**
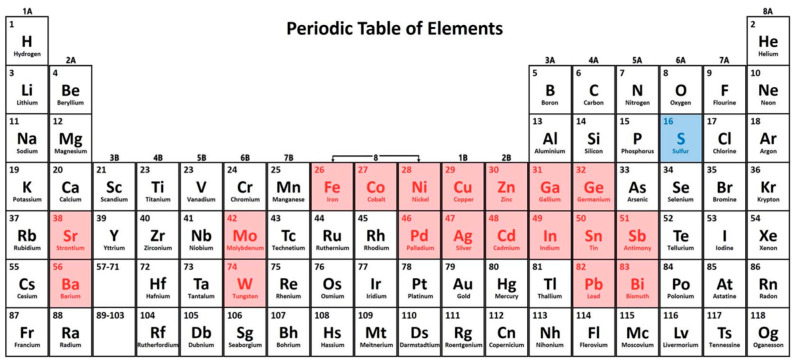
Common metallic elements used to construct binary, ternary, or quaternary sulfide photoelectrodes for PEC applications [31].

Metal sulfides are compounds formed by the combination of metal and sulfur elements. As shown in the periodic table (Figure 4), it presents the existing metal sulfides that have been employed for PEC applications. According to the number of metal elements involved, metal sulfides can be classified into binary sulfides (e.g., CdS and ZnS), ternary sulfides (e.g., ZnIn_2_S_4_ and CuInS_2_), and quaternary sulfides (e.g., Cu_2_ZnSnS_4_) [31]. Metal sulfides possess excellent light absorption properties, a broad photoresponse range, and a high efficiency of photogenerated charge carrier separation [32]. Therefore, they exhibit great potential in photocatalysis and PEC devices. For example, Wang et al. [33] proposed a PEC immunosensor based on a CdS/Bi_2_S_3_/NiS ternary sulfide heterojunction for the detection of carbohydrate antigen 125. Zhang et al. [34] developed a signal-off PEC immunosensor for the rapid and sensitive detection of cytokeratin-19 fragments, using a NiCo_2_O_4_/CdIn_2_S_4_/In_2_S_3_ heterojunction photoactive material as the sensing platform. Based on the band-aligned cascade structure and dual inhibition effect, ReS_2_@AuNPs were employed as secondary antibody labels to amplify the signal. Lin et al. [35] reported a new strategy for amplifying the photocurrent signal in an alkaline medium via the photocatalyst–electrolyte effect, utilizing a snowflake-like CdS@ZnIn_2_S_4_ heterojunction as a photosensitizer for the sensitive monitoring of prostate-specific antigen. CdS has an appropriate band gap, allowing it to absorb visible light and exhibit good photoelectric performance, but its toxicity remains a key challenge [36].

### 3.3. Graphitic Carbon Nitride

**Figure 5:**
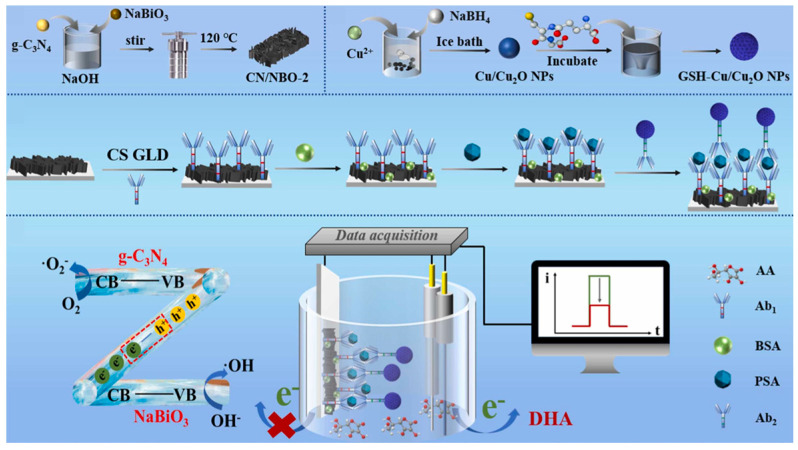
Schematic illustration of the nanozyme-mediated PEC immunoassay [38].

Graphitic carbon nitride (g-C_3_N_4_) possesses a graphite-like layered structure in which both carbon and nitrogen atoms are sp2 hybridized. Unlike the C–C bonding in graphite, all the p orbitals of the atoms in g-C_3_N_4_ overlap with each other, forming a large π conjugated system similar to that of benzene rings, which results in a highly delocalized conjugated network [37]. Owing to its unique structure and high degree of polymerization, g-C_3_N_4_ exhibits excellent thermal and chemical stability. More importantly, g-C_3_N_4_ has a bandgap of approximately 2.7 eV, endowing it with good visible-light responsiveness. In addition, it features several advantages, such as the easy availability of raw materials, simple preparation process, nontoxicity, and good biocompatibility, making it a widely studied material in photocatalysis, environmental remediation, and biosensing [26,37]. Therefore, g-C_3_N_4_ is often employed as an electrode material in PEC immunosensors. As shown in Figure 5, Wu et al. [38] developed a sandwich-type PEC immunosensor for the detection of prostate-specific antigen, in which a g-C_3_N_4_/NaBiO_3_ (CN/NBO) Z-scheme heterojunction was used as the photoelectrode, and glutathione-Cu/Cu_2_O nanozyme (GSH–Cu/Cu_2_O NPs) served as the signal amplifier. The unique charge transfer pathway within the CN/NBO heterojunction significantly promoted the separation and migration of photogenerated electron–hole pairs, resulting in a high photocurrent response. GSH–Cu/Cu_2_O NPs acted as ascorbate oxidase to catalyze the oxidation of the electron donor ascorbic acid to dehydroascorbic acid in the PEC system, thereby decreasing the photocurrent response. Benefiting from the excellent photoelectrode performance of the Z-scheme heterojunction and the signal-quenching effect of GSH–Cu/Cu_2_O NPs, the PEC immunosensor achieved an ultralow limit of detection of 5.1 fg/mL. However, the PEC performance of g-C_3_N_4_ is limited by its low electrical conductivity, rapid recombination of photogenerated charge carriers, and relatively small specific surface area [39]. To further enhance the carrier separation efficiency and visible-light absorption of g-C_3_N_4_, researchers have developed element-doped g-C_3_N_4_. For instance, Liu et al. improved charge carrier mobility through potassium–phosphorus co-doping in g-C_3_N_4_ [40].

### 3.4. Quantum Dots

**Figure 6:**
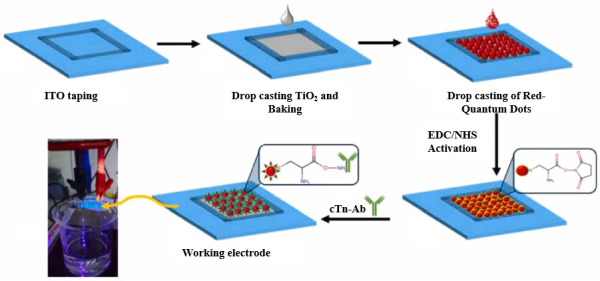
Schematic illustration of the PEC sensor based on the CdSe/CdS/ZnS core–shell quantum dot/TiO_2_ heterostructure [42].

QDs have attracted widespread attention in recent years due to their unique properties, such as tunable band gaps, quantum confinement effects, abundant surface active sites, and efficient charge transfer rates. Typically composed of elements from different groups of the periodic table, QDs exhibit a broad absorption spectrum, a low charge recombination rate, and a high photoluminescence efficiency. In PEC immunosensors, commonly used photoactive QDs include CdS, CdSe, CdTe, and ZnS. To further enhance charge separation efficiency and light absorption capability, researchers have employed strategies such as constructing composite materials and core–shell structures to optimize sensor performance [41]. Memon et al. [42] reported a PEC sensor based on a red-emitting CdSe/CdS/ZnS core–shell quantum dot/TiO_2_ heterostructure for detecting the cardiac biomarker troponin I (cTnI). As shown in Figure 6, –COOH functionalized CdSe/CdS/ZnS core–shell QDs/TiO_2_ were first activated using carbodiimide coupling agents. The antibodies of cTnI were then immobilized on the functionalized heterostructure to capture the cTnI protein. In this system, the CdSe/CdS/ZnS quantum dot layer facilitated the separation of photoexcited electrons and holes, thereby enhancing the stability of the photocurrent. The sensor exhibited a detection range from 10 pg/mL to 0.2 ng/mL for cTnI. This study demonstrated the successful application of a CdSe/CdS/ZnS core–shell quantum dot/TiO_2_-based PEC sensor for early diagnosis of cardiac diseases through cTnI detection. However, the synthesis of eco-friendly QDs remains challenging because of the reliance on toxic solvents and costly precursor salts [43].

### 3.5. Novel Photoactive Materials

Although traditional photoactive materials such as metal oxides, metal sulfides, and quantum dots have mature preparation processes, they generally suffer from fixed band gaps, poor electron transport capabilities, and certain toxicity issues in some materials (e.g., CdS, CdSe), which limit their further application in biomedical detection. To overcome these shortcomings, new materials such as metal-carbon/nitride compounds, bismuth-based oxyhalides, metal–organic frameworks, and perovskites have attracted widespread attention due to their tunable band gaps, high electron mobility, large specific surface area, and environmental friendliness. In addition, novel materials like covalent organic frameworks can also be combined with zinc-air battery devices to construct self-powered PEC sensing platforms, providing new directions for achieving high stability and wearability in sensors [44,45].

**Figure 7:**
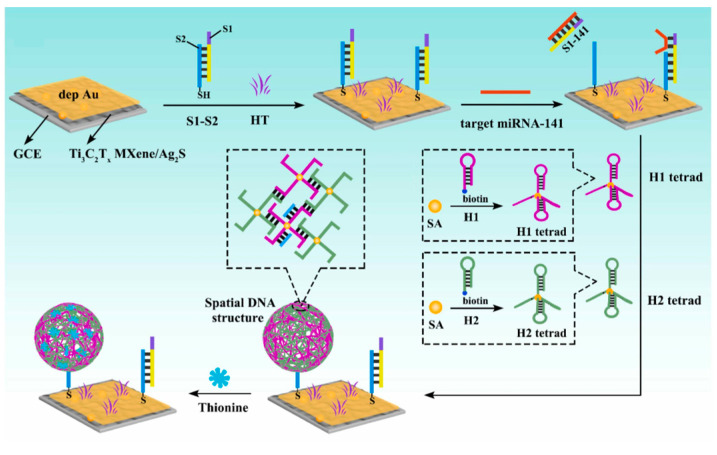
Preparation of construction steps of the biosensor [49].

Metal carbides/nitrides known as MXenes represent a new class of two-dimensional materials, generally described by the formula M_n+1_X_n_T_x_ (*n* = 1–3), where M denotes an early transition metal, X corresponds to carbon or nitrogen, and T represents surface terminations such as –O, –OH, or –F [46]. As environmentally friendly materials with excellent metallic conductivity, 2D layered MXenes possess high electron transport capability, low bandgap, large specific surface area, and a low absorption coefficient, showing great potential in energy storage, PEC systems, and related fields [47,48]. As shown in Figure 7, Huang et al. [49] prepared a novel photoactive material, Ti_3_C_2_T_x_ MXene/Ag_2_S, exhibiting strong photocurrent signals for constructing an ultrasensitive PEC biosensor for detecting target miRNA-141. Ti_3_C_2_T_x_ MXene, with its outstanding metallic conductivity, shortened the electron transport pathway, significantly enhanced the separation efficiency of electron–hole pairs, and boosted the photocurrent response of the Ti_3_C_2_T_x_ MXene/Ag_2_S nanocomposite. MXenes also face biocompatibility challenges in biological applications, as their sharp edges may cause mechanical damage to cells and possess the ability to induce oxidative stress in cells [50].

Bismuth oxyhalides (BiOX, where X = Cl, Br, or I) are a new class of bismuth-based photocatalysts characterized by their layered structures. To date, many interesting and significant findings have been achieved regarding BiOX photocatalysts [51]. Wang et al. [52] developed a liposome-assisted amplification PEC immunoassay based on ultrathin mesoporous BiOCl nanosheets for highly selective and sensitive detection of carcinoembryonic antigen. Chang et al. [53] successfully fabricated a novel label-free PEC immunosensor based on Bi_2_WO_6_/BiOBr nanocomposites for detecting prostate-specific antigen in human serum. Wang et al. [54] constructed a PEC immunosensor for neuron-specific enolase detection using ITO/BiVO_4_/BiOI/Ag_2_S as the photoanode and ITO/CuInS_2_ as the photocathode. The clinical translation of bismuth-based oxyhalides still faces challenges. Firstly, there is a lack of standardized biocompatibility testing and comparative systems. Secondly, research on their genotoxicity and the mechanisms by which they induce apoptosis remains insufficient [55].

In addition to the aforementioned inorganic photoactive materials, organic semiconductor materials such as phthalocyanines and porphyrins have also gradually become research hotspots [15]. Cheng et al. [56] developed a PEC immunosensor for carcinoembryonic antigen detection using anthocyanin-sensitized poly-5-indolecarboxylic acid nanofibers as the photoactive material. Furthermore, the combination of multiple materials can achieve synergistic improvements in light absorption range, carrier separation efficiency, and stability, thereby significantly enhancing sensing performance. The design materials and performance comparisons of various PEC sensors are summarized in Table 1.

## 4. Signal Amplification Strategies

### 4.1. Construction of Heterojunctions

The construction of heterojunctions, as one of the most effective strategies to enhance PEC sensing performance, mainly includes five types: Type-II heterojunctions, Z-scheme heterojunctions, Schottky junctions, S-scheme heterojunctions, and multicomponent heterojunctions.

**Figure 8:**
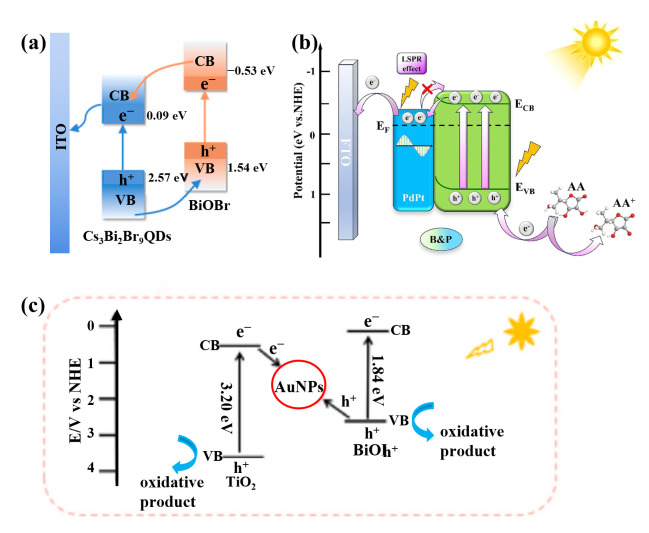
(**a**) The carrier transfer mechanism of the Type-II heterojunction [57], (**b**) the charge transfer mechanism of the Schottky junction [61], (**c**) possible charge transfer pathways in Z-scheme heterojunctions [59].

Type-II heterojunctions achieve spatial separation of electrons and holes through staggered band alignment. As shown in Figure 8a, Li et al. [57] constructed a Cs_3_Bi_2_Br_9_ QDs/BiOBr Type-II heterojunction. Under light irradiation, the VB electrons in both semiconductors were excited to their respective CB. Since the CB potential of Cs_3_Bi_2_Br_9_ QDs is 0.09 eV and that of BiOBr is −0.53 eV, the photogenerated electrons are transferred from the CB of BiOBr to the CB of Cs_3_Bi_2_Br_9_ QDs. Conversely, the VB potential of Cs_3_Bi_2_Br_9_ QDs is 2.57 eV, while that of BiOBr is 1.54 eV, resulting in the transfer of photogenerated holes from the VB of Cs_3_Bi_2_Br_9_ QDs to the VB of BiOBr, thereby achieving effective charge carrier separation. Z-scheme heterojunctions, inspired by the natural “Z-scheme” electron transfer mechanism in photosynthesis, realize efficient charge-carrier separation while maintaining strong redox ability [58]. Under light illumination, both semiconductors generate electrons and holes independently; the electrons in the CB of one semiconductor recombine with the holes in the VB of the other at the interface. This electron transfer pathway effectively reduces recombination probability and prolongs carrier lifetime. Xin et al. [59] proposed a Z-scheme TiO_2_-Au-BiOI PEC sensing platform for cyst detection. As shown in Figure 8c, Au nanoparticles (AuNPs) acted as mediators to promote the formation of the Z-scheme TiO_2_-Au-BiOI heterojunction. The built-in electric field within the Z-scheme heterostructure significantly suppressed the recombination of photogenerated electron-hole pairs and enhanced the sensitivity of the sensor. When semiconductor materials are combined with metals, a Schottky junction is formed at the interface. The resulting Schottky barrier can inhibit electrons or holes from flowing back from the metal to the semiconductor, thus suppressing electron–hole recombination [60]. As shown in Figure 8b, Zhang et al. [61] constructed a typical Schottky junction by coupling Bi_2_S_3_ nanorods with porous PdPt bimetallic nanospheres to establish an ultrasensitive PEC immunosensing platform for cTnI detection. The experimental results demonstrated that the Schottky barrier formed between PdPt and Bi_2_S_3_ effectively prevented electrons from flowing back from the metal to the semiconductor, promoting the rapid separation and transfer of photogenerated charge carriers.

**Figure 9:**
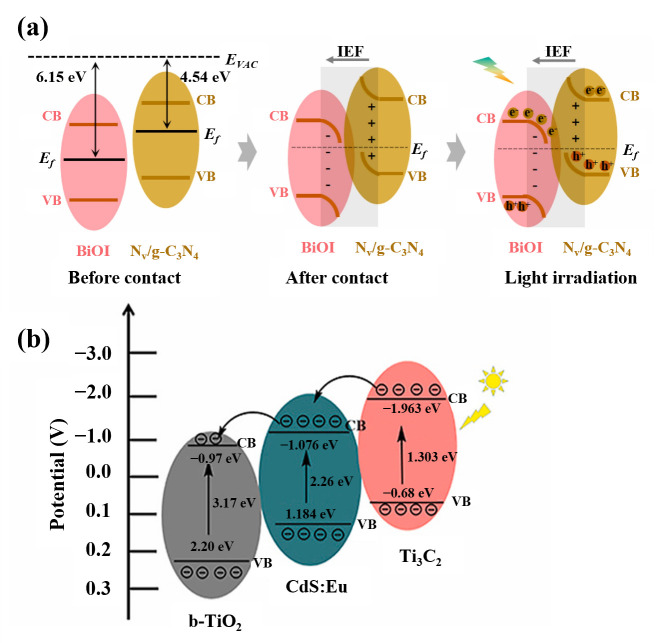
(**a**) Directional charge transfer on BiOI@Nv/g-C_3_N_4_ with the impact of internal electric field towards S-scheme heterojunction [62], (**b**) schematic diagram of the band structure of b-TiO_2_/CdS:Eu/Ti_3_C_2_ [63].

The construction of an S-scheme heterojunction requires two semiconductor materials with well-matched band structures. As shown in Figure 9a, when BiOI comes into close contact with Nv/g-C_3_N_4_, the electrons in Nv/g-C_3_N_4_, which have a higher Fermi level (−3.35 eV), will migrate toward BiOI with a lower Fermi level (−2.07 eV) along the direction of the built-in electric field in the dark. With the accumulation of electrons, the band edges of BiOI bend downward, while those of Nv/g-C_3_N_4_ bend upward due to electron depletion. Under light irradiation, the photogenerated electrons in the CB of BiOI migrate and recombine with the holes in the VB of Nv/g-C_3_N_4_ driven by Coulomb forces, thereby achieving efficient carrier separation and transfer. Therefore, an efficient S-scheme heterojunction structure was successfully constructed in the BiOI@Nv/g-C_3_N_4_ composite system [62]. Multicomponent heterojunctions can further enhance photoelectric conversion and photocurrent response through the synergistic effect of multiple photoactive materials. As shown in Figure 9b, Liu et al. [63] developed a novel PEC biosensor based on a b-TiO_2_/CdS:Eu/Ti_3_C_2_ heterojunction for ultrasensitive detection of miRNA-21. These strategies provide diverse approaches for constructing high-performance photoactive systems in PEC sensors.

### 4.2. Localized Surface Plasmon Resonance

**Figure 10:**
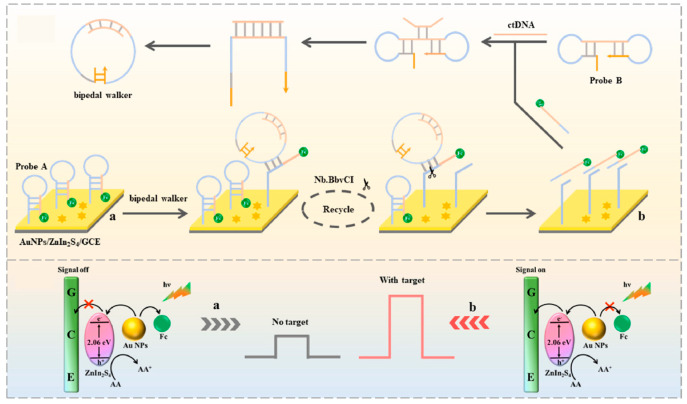
Illustration of the proposed PEC biosensor for the detection of ctDNA [66].

Localized surface plasmon resonance (LSPR) refers to the phenomenon in which the surface free electrons of metal nanoparticles (such as Au, Ag, Pt, and Pd) collectively oscillate at a specific frequency when interacting with incident light. The LSPR effect of metal nanoparticles can significantly enhance the light absorption efficiency of photoelectric materials in the visible region and generate hot electrons, thereby improving the performance of PEC immunosensors [64,65]. For example, Cui et al. [66] developed a PEC biosensor based on ZnIn_2_S_4_@AuNPs and a circular bipedal DNA walker for the signal detection of circulating tumor DNA (Figure 10). In this sensor, due to the LSPR effect of AuNPs, high-energy hot electrons are generated under visible light irradiation. These hot electrons can rapidly transfer to the CB of ZnIn_2_S_4_, thereby significantly enhancing the photoelectric signal.

### 4.3. Electron Donors/Acceptors

Electron donors and acceptors act as efficient hole or electron trapping reagents and play a crucial role in PEC biosensors. Upon light irradiation, semiconductor materials generate photogenerated electron-hole pairs, which tend to recombine easily, leading to the attenuation of the photocurrent signal. Electron donors can rapidly supply electrons to fill photogenerated holes, while electron acceptors can efficiently capture photogenerated electrons. This trapping effect effectively prolongs the lifetime of charge carriers and significantly enhances the separation efficiency of photogenerated electrons and holes. For example, Wang et al. [67] reported a novel concept for constructing PEC sensors based on the interaction between photoexcited electrons in QDs and electron acceptors. The study found that benzoquinone can act as an efficient electron acceptor for photoexcited CdS QDs, thereby blocking the electron transfer from CdS to the indium tin oxide electrode and resulting in a decreased photocurrent.

### 4.4. Defect Construction

The light absorption capability, charge carrier separation efficiency, and surface reaction activity of photoactive materials are key factors determining their sensing performance. Defect engineering is an important strategy for material regulation and mainly includes four types: elemental doping, vacancy defects, surface sensitization, and morphological and structural regulation [15]. Elemental doping involves incorporating various elements into photoactive materials to modulate the bandgap structure, enhance visible light absorption, and promote charge carrier separation. For instance, Liu et al. [68] doped a nitrogen-rich carbon layer to facilitate Z-scheme interfacial carrier transfer in TiO_2_/ZnIn_2_S_4_ heterojunctions, thereby improving photocatalytic hydrogen evolution performance. Vacancy defects utilize anion or cation vacancies to regulate the band structure. Cui et al. [69] synthesized oxygen-vacancy-regulated titanium dioxide nanotube arrays (Ov-TNTs) with high PEC activity for the detection of tetracycline hydrochloride. The synergistic effect between oxygen vacancies and Ti^3+^ led to a narrower bandgap in Ov-TNTs, allowing for the generation of more photogenerated charge carriers under light irradiation. Surface sensitization expands the light response range and enables signal amplification by modifying QDs, dyes, or noble metal nanoparticles. Zhu et al. [70] prepared phosphate-functionalized Pt/TiO_2_ as an ideal photoactive material and, combined with a Ru(bpy)_3_^2+^ sensitization strategy, constructed a split-type PEC aptasensor for detecting adenosine deaminase activity. Morphological and structural regulation improves PEC performance by controlling the material’s dimensionality and thickness. Wang et al. [71] fabricated three-dimensional, through-hole porous Ta_3_N_5_ photoanodes with precisely controlled thickness via constant-current anodization followed by NH_3_ nitridation. The results showed that a Ta_3_N_5_ thickness of 900 nm yielded optimal light absorption and charge separation efficiency. In summary, defect engineering plays a crucial role in enhancing the performance of photoactive materials, providing new design concepts and theoretical guidance for constructing high-sensitivity and high-stability PEC biosensors.

To clearly illustrate the characteristics of each strategy, Table 2 summarizes the advantages and disadvantages of the four signal amplification strategies. Future research trends will lean toward the multivariate coupling and synergistic design of these strategies, such as combining heterojunction construction with LSPR effects to further enhance the photocurrent signal [72].

## 5. Research Progress of PEC Biosensors

### 5.1. PEC Immunosensor

**Figure 11:**
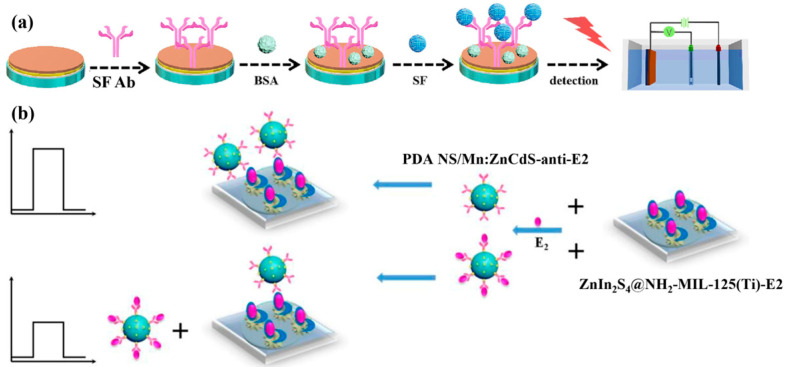
(**a**) Schematic illustration of the fabrication process for the PEC immunosensor [48], (**b**) fabrication procedure of a PEC immunosensor for detection of estradiol [78].

As an important category of PEC biosensors, PEC immunosensors can be classified into label-free and labeled types based on whether antibodies or antigens are labeled [77]. Label-free PEC immunosensors operate directly through the specific interaction between antigens and antibodies without the need for any labels, and the signal intensity is positively correlated with the concentration of the target analyte in the sample. Zhou et al. [48] employed a ZnO-MXene/Ag_2_S composite as the photoactive material to develop a visible/near-infrared light-driven PEC sensing platform. As shown in Figure 11a, in this sensor, a taxon-specific anti-sericin monoclonal antibody was used as the immunoprobe, targeting the analyte to the electrode surface through specific antibody–antigen interactions.

Labeled PEC immunosensors operate by detecting a labeled signal, in which detectable labels (such as enzymes or nanomaterials) are conjugated to antigens or antibodies. Since the labels can amplify the detection signal, labeled immunosensors generally exhibit higher sensitivity than their label-free counterparts. Depending on the sensing strategy, labeled PEC immunosensors can be further divided into competitive and sandwich types [77]. The working principle of competitive PEC immunosensors is based on the competition between labeled antigens (tracers) and unlabeled antigens (analytes) for the limited antigen-binding sites on the antibody. As shown in Figure 11b, Yan et al. [78] accurately detected free estradiol in standard samples based on the photocurrent variation resulting from the competitive binding between the polydopamine nanosphere/Mn:ZnCdS-estradiol antibody complex and either free estradiol or estradiol immobilized on the ZnIn_2_S_4_@NH_2_-MIL-125(Ti) substrate.

**Figure 12:**
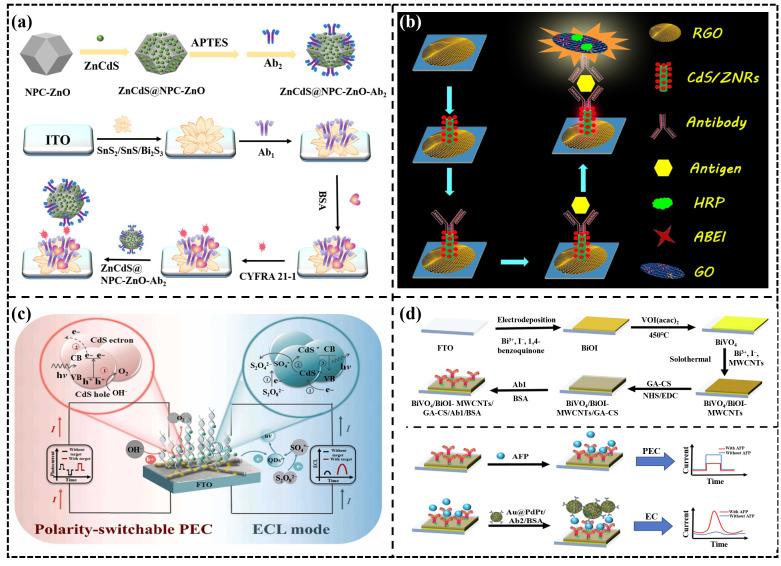
(**a**) Schematic illustration of the designed dual-signal amplification PEC immunosensor [79], (**b**) schematic representation of the fabrication and assay procedure of PEC immunosensor [80], (**c**) schematic illustration of the construction of the PEC-ECL biosensor for TP53 gene detection [81]. The asterisk (*) denotes the excited state. (**d**) alpha-fetoprotein detection process of the PEC-EC immunosensor [82].

A sandwich-type PEC immunosensor is composed of a capture antibody (Ab1), a target antigen, and a labeled detection antibody (Ab2). Due to its excellent sensitivity and stability, this method has been widely applied in the field of immunoassays. The sandwich configuration allows for dual signal amplification. As shown in Figure 12a, Liu et al. [79] synthesized a SnS_2/_SnS heterojunction and grew Bi_2_S_3_ in situ on its surface, which greatly enhanced the visible-light harvesting efficiency and PEC signal. Meanwhile, the ZnCdS@NPC-ZnO composite material labeled with Ab2 provided a dual signal amplification response. The sandwich-type PEC immunosensor can also provide a light source by labeling the Ab2 with luminescent materials. Natural light may cause background noise in PEC sensors. The sandwich-type configuration can label Ab2 with luminescent materials, providing a light source to reduce noise interference. As shown in Figure 12b, Ge et al. [80] constructed a PEC immunosensor based on CdS-sensitized ZnO nanorods/reduced graphene oxide (CdS/ZNRs/RGO) as the photoactive substrate and an N-aminobutyl-N-ethylisoluminol/graphene oxide@horseradish peroxidase (ABEI/GO@HRP/Ab2) chemiluminescent system as the internal excitation light source for the highly sensitive detection of cancer antigen 125. In addition, the sandwich-type configuration can provide dual signals to overcome the limitation of the traditional PEC system, which lacks an internal error-correction mechanism. Like traditional electrochemical detection, PEC sensors operate in the liquid phase, where electrodes are prone to contamination by complex matrices, which can lead to signal attenuation and even false positive results [11]. The sandwich-type configuration can provide dual signals and effectively improve accuracy and reliability through an integrated self-calibration model, thereby eliminating interference from complex matrices. As shown in Figure 12c, Li et al. [81] developed a novel bipolar switchable photoelectrochemical-electrochemiluminescence (PEC-ECL) dual-signal biosensor for the highly sensitive detection of the TP53 gene. As shown in Figure 12d, Wu et al. [82] proposed a biofunctionalized immunosensor that integrates photoelectrochemical-electrochemical (PEC-EC) techniques for the quantitative detection of alpha-fetoprotein, a liver cancer biomarker, in human blood.

### 5.2. PEC Gene Sensor

**Figure 13:**
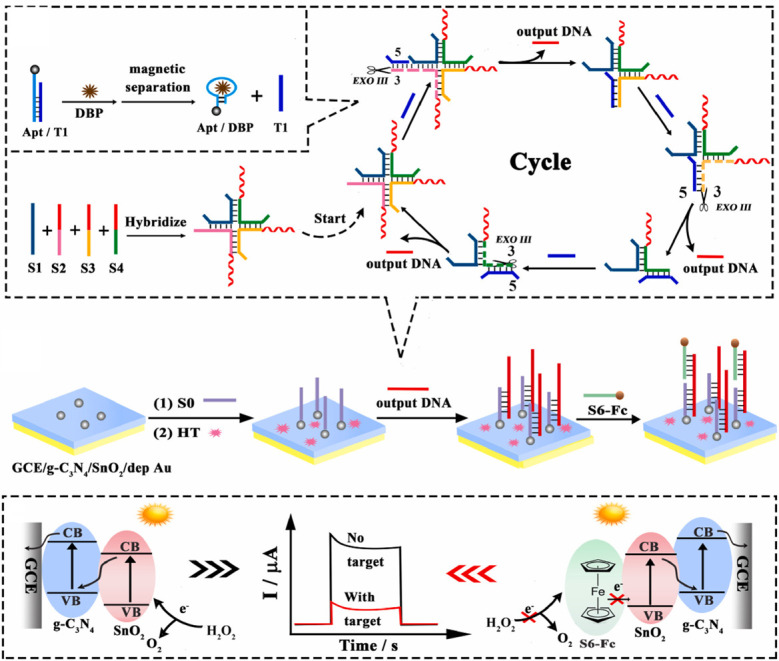
Illustrates the construction process of the PEC biosensor based on the target-induced exonuclease III-assisted cruciform DNA signal amplification strategy and its electron transfer mechanism [83].

PEC gene sensors combine the high specificity of DNA molecular recognition with the high sensitivity of PEC detection, making them an important research focus in the field of bioanalysis. Typically, such sensors use photoactive materials as substrates, onto which single-stranded DNA probes are immobilized. When the target DNA undergoes specific hybridization or binding with these probes, a change in photocurrent signal occurs, enabling quantitative detection of the target. Yang et al. [83] designed a novel signal amplification strategy based on target-induced assembly of a cruciform DNA structure to construct a highly sensitive PEC biosensor. As shown in Figure 13, after the recognition of dibutyl phthalate by the aptamer chain, a strand displacement reaction generates a simulated target (T1), which induces the assembly of four single strands (S1–S4) into a cruciform DNA structure with three recognition sites. Under the action of exonuclease III, T1 triggers cleavage and release of output DNA. Using a g-C_3_N_4_/SnO_2_ composite as the photoactive substrate, a strong initial PEC signal was obtained, and AuNPs were electrodeposited on the surface to immobilize capture DNA (S0). In the presence of dibutyl phthalate, S0 partially hybridizes with the released output DNA, followed by the binding of the ferrocene-modified strand (S6-Fc) with the output DNA. The presence of S6-Fc hinders the transfer of holes from the VB of SnO_2_ to H_2_O_2_, resulting in a decrease in photocurrent, thereby enabling the quantitative detection of dibutyl phthalate.

### 5.3. PEC Microfluidic Biosensors

**Figure 14:**
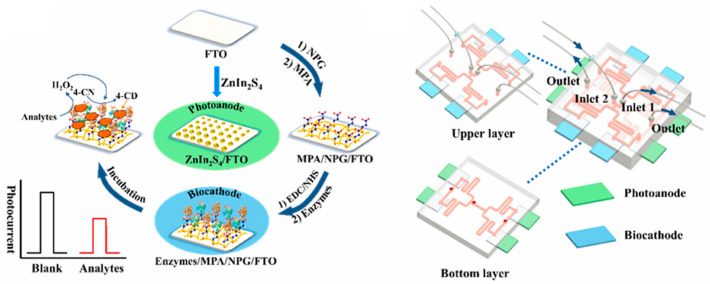
Illustrates the preparation process of the photoanode and biocathode, as well as the schematic diagram of the constructed microfluidic chip [23].

A microfluidic device, commonly referred to as a lab-on-a-chip, is a chip-based system that enables the manipulation of fluid flow and integration of multiple biochemical processes within submillimeter-scale microchannels [84]. The integrated design of microfluidics allows for the automation of many procedures that traditionally rely on manual operation, thereby improving analytical speed while offering high portability, low sample consumption, and good disposability [85]. Consequently, PEC biosensors based on microfluidic technology can achieve miniaturization and integration for tumor biomarker detection, while also providing a stable detection environment. Zeng et al. [86] developed a single-channel microfluidic PEC platform for detecting the liver cancer biomarker PIVKA-II. Du et al. [87] constructed an innovative dual-channel microfluidic PEC immunosensor for the simultaneous detection of carbohydrate antigen and cancer antigen 125. Li et al. [23] designed a multifunctional detection chip integrating microfluidic technology with a PEC sensor for the simultaneous monitoring of multiple clinical indicators, including diabetes, lactic acidosis, and diabetic ketoacidosis. As shown in Figure 14, the PEC sensor in this chip consists of two parts: a biocathode and a photoanode. The photoanode is composed of four pieces of ZnIn_2_S_4_ modified fluorine-doped tin oxide coated glass, which can generate a stable photocurrent under a certain light intensity. The biocathode comprises four FTO electrodes modified with nanoporous gold (NPG), horseradish peroxidase (HRP), and specific enzymes, including glucose oxidase, lactate oxidase, pyruvate oxidase, and D-3-hydroxybutyrate dehydrogenase. During detection, each specific enzyme catalyzes the corresponding analyte in the sample to produce H_2_O_2_. Subsequently, under the catalysis of HRP, H_2_O_2_ oxidizes 4-chloro-1-naphthol into an insulating benzo-4-chlorohexadienone, which deposits on the surface of the biocathode, leading to a decrease in photocurrent. This photocurrent variation enables the quantitative detection of the target analytes. Microfluidic technology can also integrate filtration and enrichment functions. Wang et al. [88] developed a novel filtration-electrochemical microfluidic chip capable of directly detecting and classifying breast cancer in whole blood without requiring extensive purification methods.

**Figure 15:**
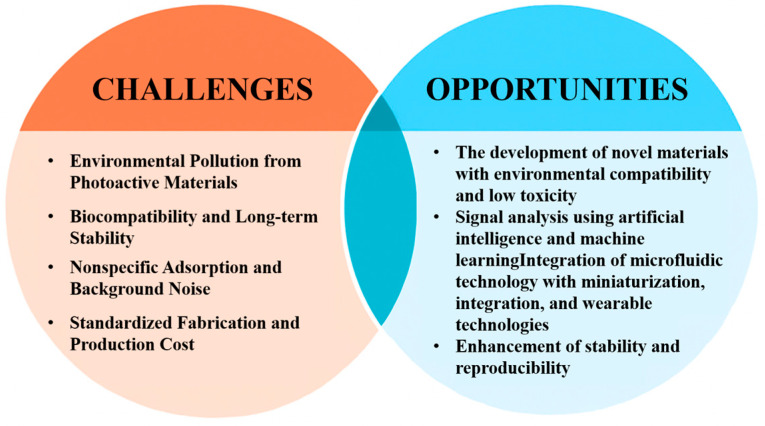
Schematic of the main challenges and opportunities of PEC sensors.

Although various PEC biosensors have demonstrated high sensitivity and selectivity under laboratory conditions, they still face multiple challenges in real sample detection and clinical translation (as shown in Figure 15). Firstly, some photosensitive materials may become unstable after repeated or prolonged use, affecting the sensor’s reproducibility and stability. Secondly, despite the excellent performance of certain materials, their high material and manufacturing costs remain a significant barrier to commercialization. Moreover, PEC signals are highly dependent on the light source, and natural light or coexisting interfering elements may lead to background noise, which in turn affects the accuracy of the results. Furthermore, the design and fabrication of PEC biosensors is complex, requiring expertise in both electrochemistry and spectroscopy, as well as considerations of photoelectrode selection, the preparation of photoactive materials, and integration with the bio-recognition elements. Different materials and processes have a significant impact on the performance of the final sensor [89]. Additionally, the standardization of PEC detection platforms is relatively low, making it difficult to ensure result comparability and achieve mass production. Some photosensitive materials (such as those containing Cd or Pb) still pose bio-safety risks and environmental pollution concerns, limiting their widespread application in clinical and environmental detection.

## 6. Conclusions

With the continuous development of photoactive material design and signal amplification strategies, PEC sensing technology has made significant progress in sensitivity, selectivity, and stability. Additionally, the introduction of immune sensor structural design, DNA-based assembly systems, nanoenzyme catalysis, and microfluidic chip technology has provided new solutions for complex sample analysis and multi-parameter detection. Although significant progress has been made, there are still some research gaps and unresolved issues. For example, the environmental pollution, biocompatibility issues, and long-term stability associated with photoactive materials (e.g., QDs and sulfides) need further improvement. Non-specific adsorption in complex biological samples and background noise caused by natural light and other factors remain technical challenges. Future research can focus on the following directions: First, the development of new eco-friendly, low-toxicity materials with good photoelectric performance to promote the environmental friendliness and sustainability of PEC sensors. Second, the integration of artificial intelligence and machine learning technologies to achieve automated signal analysis. Meanwhile, the combination of microfluidic technology can push PEC biosensors towards miniaturization, integration, and wearability, enabling convenient real-time detection. Overall, the future development of photoelectrochemical biosensors will make significant progress in improving performance, enhancing stability, reducing costs, and promoting standardized applications. This will gradually bridge the gap from laboratory research to practical applications, becoming an important technological support in fields such as precision medicine and environmental monitoring.

## Figures and Tables

**Table 1:** Summary of PEC sensor design materials and performance comparison [13].

Photoactive Material	Analyte	Linear Range	Detection Limit
TiO_2_/CdS:Mn	DNA	0.0005–50 pm	27 am
ZnO flower-rod architectures	DNA	0.00001–100 nm	3.7 fm
CdTe QDs/ZnO NSs	DNA	0.01–10 pm	0.93 fm
Bi_2_S_3_ NRs	miRNA	1–5000 fm	0.35 fm
TiO_2_-CdS:Mn and Au NPs	miRNA	1.0–10,000 fm	0.5 fm
Au-TiO_2_	prion protein	200–2000 fm	50.9 fm
phosphorylated g-C_3_N_4_ NPs	PKA	0.05–50 U/mL	0.077 U/mL
RGO-BiFeO_3_	PSA	0.001–100 ng/mL	0.31 pg/mL
BiVO_4_-RGO	PSA	10–80 ng/mL	3.0 pg/mL
Au-BiVO_4_	PSA	10–100 ng/mL	4.0 pg/mL
CdS-PAMAM film	SMMC-7721 cells	5.0 × 10^3^–1.0 × 10^7^ cells/mL	5.0 × 10^3^ cells/mL
graphene-CdS film	HeLa cells	1.0 × 10^2^–5.0 × 10^6^ cells/mL	100 cells/mL
CdSe QDs/TiO_2_	o-aminophenol	0.4–27 μm	80 nm

**Table 2:** Comparison of different signal amplification strategies.

Strategies	Advantage	Disadvantage	Performance
heterojunctions construction	enhancing carrier separation efficiency	band matching is challenging to control with precision	a 7.0-fold higher photocurrent of BiFeO_3_/g-C_3_N_4_ versus BiFeO_3_ [73]
LSPR	extending the light absorption range	high cost and reproducibility related to particle size	a 3.5-fold higher current of Ag_2_S/AuNPs versus Ag_2_S [74]
donors/acceptors	enhancing carrier separation efficiency	performance depends on donor/acceptor concentration and diffusion efficiency	The electron donor 1,4-diazabicyclo[2.2.2]octane increased the current by 23.2-fold [75]
defect construction	enhancing light absorption and carrier migration efficiency	defect concentration is difficult to control	selenium doping in In_2_S_3_ resulted in a fourfold increase in the anodic photocurrent [76]

## Data Availability

No new data were created or analyzed in this study.
